# Fermented milk improves glucose metabolism in exercise-induced muscle damage in young healthy men

**DOI:** 10.1186/1475-2891-12-83

**Published:** 2013-06-16

**Authors:** Masayo Iwasa, Wataru Aoi, Keitaro Mune, Haruka Yamauchi, Kaori Furuta, Shota Sasaki, Kazuya Takeda, Kiyomi Harada, Sayori Wada, Yasushi Nakamura, Kenji Sato, Akane Higashi

**Affiliations:** 1 Laboratory of Health Science, Graduate School of Life and Environmental Sciences, Kyoto Prefectural University, 1-5 Hangi-cho Shimogamo, Sakyo-ku, Kyoto, Japan; 2 Laboratory of Food Science, Graduate School of Life and Environmental Sciences, Kyoto Prefectural University, 1-5 Hangi-cho Shimogamo, Sakyo-ku, Kyoto, Japan

**Keywords:** *Lactobacillus helveticus*, Delayed-onset muscle damage, Inflammation, Oxidative stress, Antioxidant

## Abstract

**Background:**

This study investigated the effect of fermented milk supplementation on glucose metabolism associated with muscle damage after acute exercise in humans.

**Methods:**

Eighteen healthy young men participated in each of the three trials of the study: rest, exercise with placebo, and exercise with fermented milk. In the exercise trials, subjects carried out resistance exercise consisting of five sets of leg and bench presses at 70–100% 12 repetition maximum. Examination beverage (fermented milk or placebo) was taken before and after exercise in double-blind method. On the following day, we conducted an analysis of respiratory metabolic performance, blood collection, and evaluation of muscle soreness.

**Results:**

Muscle soreness was significantly suppressed by the consumption of fermented milk compared with placebo (placebo, 14.2 ± 1.2 score vs. fermented milk, 12.6 ± 1.1 score, *p* < 0.05). Serum creatine phosphokinase was significantly increased by exercise, but this increase showed a tendency of suppression after the consumption of fermented milk. Exercise significantly decreased the respiratory quotient (rest, 0.88 ± 0.01 vs. placebo, 0.84 ± 0.02, *p* < 0.05), although this decrease was negated by the consumption of fermented milk (0.88 ± 0.01, *p* < 0.05). Furthermore, exercise significantly reduced the absorption capacity of serum oxygen radical (rest, 6.9 ± 0.4 μmol TE/g vs. placebo, 6.0 ± 0.3 μmol TE/g, *p* < 0.05), although this reduction was not observed with the consumption of fermented milk (6.2 ± 0.3 μmol TE/g).

**Conclusion:**

These results suggest that fermented milk supplementation improves glucose metabolism and alleviates the effects of muscle soreness after high-intensity exercise, possibly associated with the regulation of antioxidant capacity.

## Background

Unaccustomed and strenuous exercise causes muscle damage that clinically presents as muscular pain and involves protein degradation and ultrastructural changes, a condition known as delayed-onset muscle damage. The exercise-induced muscle damage is caused by several factors, including mechanical stress, calcium accumulation, and oxidative stress [1-4]. It has been suggested that muscle functions, such as energy metabolism and power output, are difficult to maintain in damaged muscle. Previous studies have reported that glucose utilization as an energy substrate in whole body is decreased in muscle damage after exercise [5,6], caused by an impairment of insulin-dependent glucose uptake in the damaged muscle [5].

It has been reported that oxidative stress and certain inflammatory cytokines impair glucose uptake via inactivation of insulin signaling pathways in muscle cells [7-9]. Infiltration of phagocytes into the damaged muscle is observed after strenuous exercise and an inflammatory response is implicated in the development of delayed-onset muscle damage [2,10]. In addition, elevation of the levels of oxidative damage in cellular components is also observed in damaged muscle [1,2]. Thus, inflammatory cytokines and oxidative stress can decrease insulin-dependent glucose uptake in exercise-induced damaged muscles [11]. Therefore, we hypothesized that the decrease of glucose metabolism associated with muscle damage may be prevented by the suppression of inflammation and oxidative stress.

Fermented milk has several salutary effects, including prolonged lifespan, antihypertensive and antitumorigenic effects, and immune system regulation [12-15]. In addition, some types of fermented milk also possess anti-inflammatory and antioxidant properties [16-19]. Previously, we have shown that *Lactobacillus helveticus*–fermented milk prevents muscle damage induced by acute exercise via activation of antioxidative enzymes of skeletal muscle in an animal study [20], suggesting that fermented milk may prevent the impairment of glucose metabolism associated with muscle damage. Thus, the purpose of this study is to investigate the effect of fermented milk supplementation on glucose metabolism in damaged muscle after acute resistance exercise in humans.

## Methods

### Subjects

Eighteen healthy young men who were not have the habituated to a regular exercise regimen were recruited to participate in this study. The characteristics of the subjects were follows: age, 21.6 ± 0.8 yr; height, 171.1 ± 1.5 cm; body weight, 59.9 ± 1.5 kg; body mass index, 20.5 ± 0.4 kg/m^2^; and body fat, 16.2 ± 0.8%. All subjects were free of signs, symptoms, and history of any overt chronic disease. None of the participants had a history of smoking and none were currently taking any medications or dietary supplements. This study was approved by the Ethics Committee of Kyoto Prefectural University, and all subjects signed a consent form after reading the design and protocol of the study.

### Study design

The subjects participated in three trials of the study: rest with placebo intake (rest), exercise with placebo intake (placebo), and exercise with fermented milk intake (fermented milk) in a repeated-measures experimental design. These trials were performed in a random order by a counter-balanced design and were separated by at least six weeks in any individual subject to avoid the biasing of muscle damage. Subjects were also asked to refrain from caffeine and alcohol ingestion 24 h before each trial. Food intake was recorded on the day before the trial and the diet was repeated before each successive individual treatment.

### Examination beverage

*Lactobacillus helveticus*–fermented milk (Amiel S®, Calpis Co., Ltd., Tokyo, Japan) was used in the fermented milk trial. An equivalent dose of unfermented milk, with adjusted contents of protein (1.1%), fat (0%), carbohydrate (3.6%), and pH (3.75) to be equivalent with that of fermented milk, was used as a placebo beverage. Subjects consumed 200 mL of each beverage 3 times before and after exercise by the double-blinded method; therefore, they totally took energy: 102 kcal, protein: 6.6 g, fat: 0.0 g, and carbohydrate: 21.6 g / 600 mL.

### Experiment schedule

**Figure 1:**
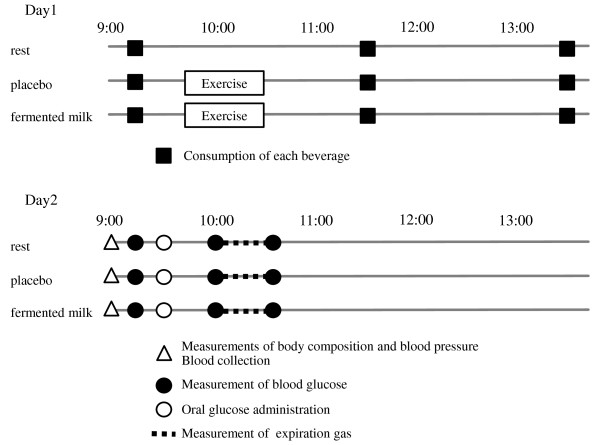
Schematic illustration of the experimental schedule.

On the first experiment day of each trial, subjects came to laboratory at 9:00, sat on a chair, and were made to rest. Subsequently, the test beverage was consumed at 9:15. Blood pressure and heart rate were monitored with a humerus sphygmomanometer (EW3100, Panasonic Electric Works Co., Ltd., Osaka, Japan). In the rest trial, subjects refrained from exertional activity, and were maintained in a state of rest. In the placebo and fermented milk trials, resistance exercise was performed from 30 min after beverage consumption. Blood lactate was measured by a blood test after a fingerstick prick at pre- and post-exercise periods. Afterwards, the test beverage was consumed again at 11:30 and 13:30. The subjects were asked to not to eat or drink anything except for water from 22:00 to the measurement of the next morning. On the second day of the study, subjects collected a sample from the first urine flow in the morning (urine accumulated overnight), returned to the laboratory at 9:00 while maintaining their fast, sat on the chair, and were made to rest. Subsequently, the body composition and blood pressure were measured, and blood was collected from the antecubital vein. Glucose solution containing 75 g glucose (Trelan®–G75, Ajinomoto Pharmaceuticals Co. Ltd., Tokyo, Japan) was orally consumed at 9:30, and the expiration gas was measured from 10:00 for 30 min in the supine position. Subsequently, subjective muscle pain in pectoralis major, quadriceps, and gluteus maximus was evaluated by palpation and movement (butterfly and squat) using the visual analog scale (VAS). The VAS was used to examine the level of muscle pain. Subjects were asked to indicate the intensity of perceived pain for each muscle part on a 100-mm horizontal line. The left side stated “having no pain”, while the right side stated “having max pain”. The total soreness value was calculated by adding the soreness values on 3 muscle parts. Blood glucose was measured by a blood test after a fingerstick prick before, 30 min after, and 60 min after oral glucose administration. A schematic illustration of the experimental schedule was shown in Figure 1.

### Exercise procedure

After warm-up with a bicycle ergometer for 5 min and stretching, subjects undertook resistance exercise for 45 min. The resistance exercise was composed of leg and bench presses using a compound-type resistance training machine (Senoh Ltd., Chiba, Japan). Five sets of leg and bench presses were performed at a strength of 70–100% with a 12-repetition maximum (RM: maximum number of occurrence). This strength was determined using the methods of Drummond et al. [21]. All subjects performed 10 repetitions at the load of 100% 12RM in 1–3sets and then the load of 70% 12RM in 4–5sets. The exercises were repeated at a pace of one repetition every 3 sec, with a 2-min interval between sets.

### Indirect metabolic performance

Oxygen consumption (VO_2_) and carbon dioxide production (VCO_2_) were measured using a breath-by-breath respiromonitor system (MetaMax 3B, Cortex, Leipzig, Germany). The respiratory quotient (RQ) and substrate utilization were calculated from the level of VO_2_ and VCO_2_, as described previously [22].

### Blood and urine parameters

Blood lactate and glucose were measured using simple measuring instruments (Lactate Pro, GluTest; Arkray, Inc., Kyoto, Japan). The analysis of neutral fat, cholesterols (LDL-cholesterol, HDL-cholesterol, and total cholesterol), free fatty acid, high sensitivity C-reactive protein (hsCRP), and creatine phosphokinase (CPK) in serum was entrusted to FALCO Biosystems Corporation (Kyoto, Japan). Tumor necrosis factor α (TNF-α) and carbonyl protein levels in the serum were measured by using enzyme-linked immunosorbent assay (ELISA) kit (TNF-α: R&D Systems, MN, USA; carbonyl protein: BioCell, Auckland, NZ). Oxygen radical absorbance capacity (ORAC), a marker that reports antioxidant capacity, was measured by using the methods of Watanabe et al. [23]. The concentration of 8-hydroxydeoxyguanosine (8-OHdG), a marker of DNA oxidative damage, was measured using an ELISA kit (Japan Institute for the Control of Ageing, Fukuroi City, Shizuoka, Japan) on the gathered urine, and the total amount of 8-OHdG was calculated by the volume of urine. Moreover, measurement of the creatinine was requested (FALCO Biosystems Ltd.) and used to correct the amount of 8-OHdG. These parameters cannot be analyzed for several subjects either because sample volume was insufficient or because subjects forgot to collect their urine samples.

### Statistical analysis

All data were shown by mean value ± standard error. The significance level was assumed to be 5% (*p* < 0.05). The repeated-measures analysis of variance (ANOVA) was used to compare the date the 3 trials. In the index of 2–3 collection points per trial, such as blood glucose and lactate, a 2-way repeated-measures ANOVA was used. If ANOVA indicated a significance difference, a Tukey-Kramer test was used to determine the significance of the differences between mean values. Paired t-tests were used in the comparisons of muscle soreness and blood lactate between the two trials.

## Results

### Muscle damage parameters and blood lactate

The level of blood lactate was markedly increased immediately after exercise, but there were no significant differences in blood lactate levels between the placebo and fermented milk trials. On the day following the exercise, serum CPK was significantly elevated in the placebo trial compared with the rest trials (*p* < 0.01), although serum CPK of the fermented milk trial showed a tendency to decrease compared with that of the placebo trial (Table 1). Muscle soreness of pectoralis major was significantly suppressed by the consumption of fermented milk compared with that of placebo in the evaluation by palpation (*p* < 0.05) (Table 1). Muscle soreness of quadriceps and gluteus maximus also showed a tendency to be reduced by the consumption of fermented milk compared with that of placebo (Table 1). The total score of muscle soreness in the three parts was significantly suppressed by the consumption of fermented milk compared with that of placebo (*p* < 0.05) (Table 1). In the evaluation by movements, the level of muscle soreness was also significantly suppressed (date not shown).

**Table 1:** Comparison of muscle damage parameters

	**Rest**	**Placebo**	**Fermented milk**
CPK (IU·L^-1^)	95.7 ± 7.2	192.8 ± 26.9**	152.3 ± 14.7*
Muscle soreness			
Pectoralis major (score)	N.D	5.2 ± 0.5	4.3 ± 0.5^#^
Quadriceps (score)	N.D	3.3 ± 0.5	3.0 ± 0.5
Gluteus maximus (score)	N.D	5.6 ± 0.5	5.3 ± 0.5
Total score (score)	N.D	14.2 ± 1.2	12.6 ± 1.1^#^

Values are represented as mean ± standard error for 18 subjects. *Statistically significant differences were at the level of *p* < 0.05 vs. rest. **Statistically significant differences at the level of *p* < 0.01 vs. rest. ^#^Statistically significant differences at the level of *p* < 0.05 vs. placebo. CPK, creatine phosphokinase; N.D., Not Detected. The trials analyzed include: rest, rest with placebo intake; placebo, exercise with placebo intake; fermented milk, exercise with fermented milk intake.

### Indirect metabolic performance

**Figure 2:**
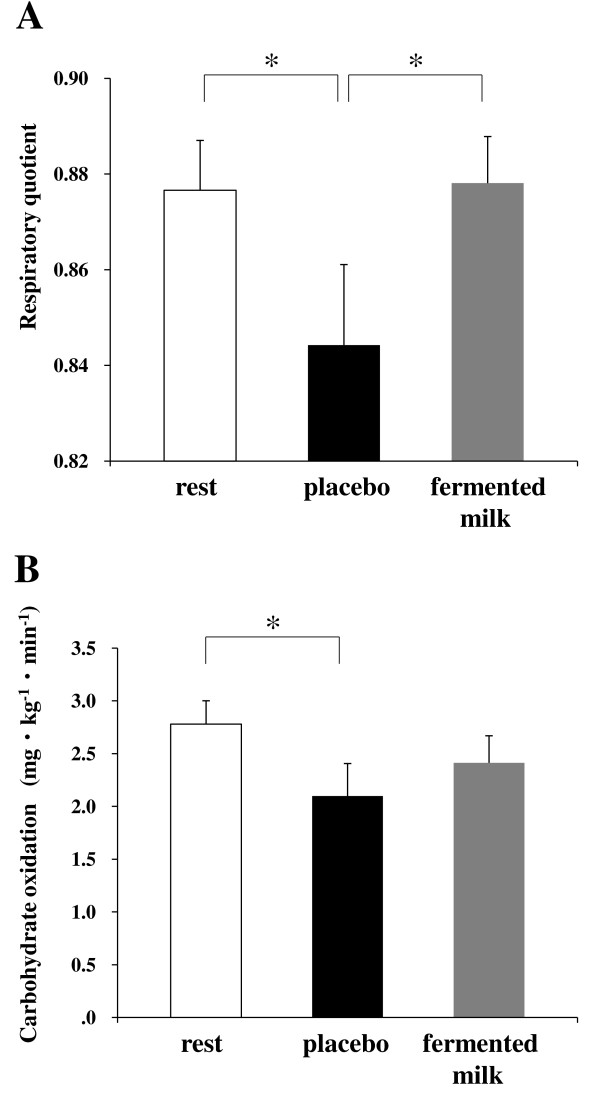
**Comparison of respiratory metabolic performance.** Respiratory quotient (**A**) and carbohydrate oxidation (**B**) were calculated using oxygen consumption and carbon dioxide production and were compared among the mean values of the three trials for 30 min after glucose administration. The trials analyzed include: rest, rest with placebo intake; placebo, exercise with placebo intake; fermented milk, exercise with fermented milk intake. Values are represented as mean ± standard error for 18 subjects. *, Statistically significant differences were at the level of *p* < 0.05.

The RQ and carbohydrate oxidation were compared among the mean values of the three trials for 30 min after glucose administration. The RQ was significantly decreased in the placebo trial compared with the rest trial (control, 0.88 ± 0.01 vs. placebo, 0.84 ± 0.02, *p* < 0.05), although this decrease was negated by the consumption of fermented milk (0.88 ± 0.01) (Figure 2A). Consistent with RQ, carbohydrate oxidation was significantly decreased in the placebo trial compared with the rest trial (control, 2.78 ± 0.02 mg/kg/min vs. placebo, 2.10 ± 0.31 mg/kg/min, *p* < 0.05), although this decrease was not found in the fermented milk trial (2.41 ± 0.26 mg/kg/min) (Figure 2B). On the other hand, fat oxidation and oxygen consumption did not significantly differ among the trials of the study (data not shown).

### Blood glucose and serum lipids

Blood glucose levels were increased after oral glucose administration, although the change was not deemed to be statistically significant among the three trials at fast, 30 min, and 60 min (Table 2). Levels of LDL-cholesterol, HDL-cholesterol, total cholesterol, triglyceride, and free fatty acids were not significantly changed among three trials (Table 3).

**Table 2:** Comparison of blood glucose level after oral glucose administration

	**Rest**	**Placebo**	**Fermented milk**
Fasting (mg·dL^-1^)	89.1 ± 1.8	86.7 ± 2.4	90.3 ± 2.1
30 min (mg·dL^-1^)	147.9 ± 5.5	147.8 ± 5.9	156.7 ± 4.9
60 min (mg·dL^-1^)	121.1 ±5.6	121.7 ±5.9	132.4 ± 7.0

Values are represented as mean ± standard error for 18 subjects. The trials analyzed include: rest, rest with placebo intake; placebo, exercise with placebo intake; fermented milk, exercise with fermented milk intake.

**Table 3:** Comparison of serum lipids

	**Rest**	**Placebo**	**Fermented milk**
LDL Cholesterol (mg·dL^-1^)	83.5 ± 5.1	88.4 ± 5.4	91.0 ± 5.5
HDL Cholesterol (mg·dL^-1^)	57.4 ± 3.4	58.8 ± 3.1	59.2 ± 3.1
Total Cholesterol (mg·dL^-1^)	157.9 ± 7.1	163.1 ± 5.3	163.6 ± 6.6
Triglycerides (mg·dL^-1^)	85.3 ± 11.9	79.5 ± 14.2	67.2 ± 6.5
Free fatty acids (mEq·L^-1^)	0.38 ± 0.04	0.47 ± 0.06	0.38 ± 0.05

Values are represented as mean ± standard error for 18 subjects. The trials analyzed include: rest, rest with placebo intake; placebo, exercise with placebo intake; fermented milk, exercise with fermented milk intake.

### Inflammation and oxidant stress parameters

**Figure 3:**
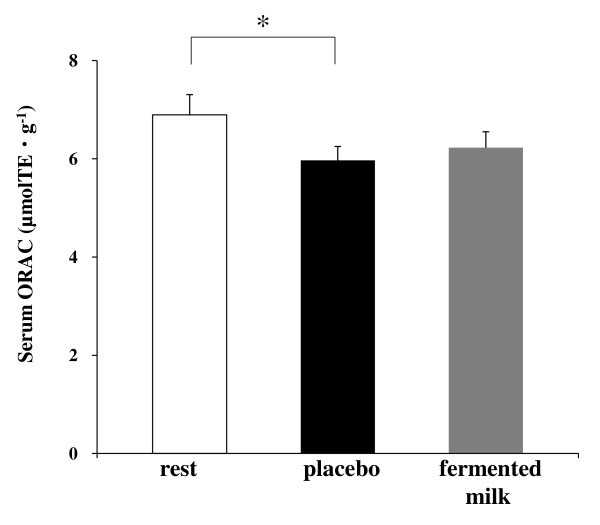
**Comparison of serum oxygen radical absorbance capacity (ORAC).** The trials analyzed: rest, rest with placebo intake; placebo, exercise with placebo intake; fermented milk, exercise with fermented milk intake. Values are represented as mean ± standard error for 13 subjects. *, Statistically significant differences were at the level of *p* < 0.05.

Serum hsCRP was not significantly changed among the trials of the study, although it showed a tendency of increasing in the placebo trial, but not in the fermented milk trial (Table 4). Serum TNF-α and carbonyl protein levels were not significant changed among the trials of the study, nor were urine 8-OHdG levels (Table 4). However, the exercise trial reported significantly decreased levels of serum ORAC compared with the rest trials (control, 6.9 ± 0.4 μmolTE/g vs. placebo, 6.0 ± 0.3 μmolTE/g, *p* < 0.05), although this decrease was not observed in the fermented milk trial (6.2 ± 0.3 μmolTE/g) (Figure 3).

**Table 4:** Comparison of inflammatory factors and oxidant stress markers

	**Rest**	**Placebo**	**Fermented milk**
Serum hsCRP (ng·mL^-1^)	88.9 ± 11.7	113.6 ± 22.1	79.3 ± 10.6
Serum TNF-α (pg·mL^-1^)	0.161 ± 0.002	0.166 ± 0.001	0.170 ± 0.004
Serum carbonyl protein (nmol·mg^-1^)	0.086 ± 0.003	0.089 ± 0.004	0.096 ± 0.003
Urine 8-OHdG (μg)	1.82 ± 0.16	1.70 ± 0.16	1.72 ± 0.19

Values are represented as mean ± standard error for 12 subjects (hsCRP and TNF-α), 18 subjects (carbonyl protein), and 13 subjects (8-OHdG). The trials analyzed include: rest, rest with placebo intake; placebo, exercise with placebo intake; fermented milk, exercise with fermented milk intake. hsCRP, high sensitivity C-reactive protein; TNF-α, tumor necrosis factor alpha; 8-OHdG, 8-hydroxydeoxyguanosine.

## Discussion

The present study revealed the following main findings: 1) parameters of muscle damage were elevated on the day following acute resistance exercise; 2) the decrease of carbohydrate oxidation along with RQ was observed with exercise; and 3) the muscle soreness and metabolic changes were mitigated by the consumption of *Lactobacillus helveticus*–fermented milk in pre- and post-exercise. Previously, it had been unclear whether dietary intervention can improve metabolic impairment after muscle-damaging exercise. Our observations primarily demonstrate that dietary fermented milk improves the impairment of glucose metabolism associated with exercise-induced muscle damage in humans. Previously, consumption of milk (unfermented) partially attenuates the muscle damage [24]; therefore, the placebo trial, which used unfermented milk, may have also suppressed muscle damage to some extent. However, our results showed that fermented milk is more effective than milk.

Generally, it is well-known that a single bout of exercise elevates glucose uptake for a period of time post-exercise [25-27]. However, we have shown that insulin-mediated glucose uptake in muscle is decreased by muscle-damaging exercises, but not by non-muscle-damaging exercises [5]. Therefore, the decreases of carbohydrate oxidation and respiratory quotient in the present study are suspected to be caused by insulin-dependent glucose uptake in damaged muscle. This decrease of glucose uptake is presumably due to a reduction of glucose transporter protein 4 (GLUT4) translocation via the insulin signaling pathway, which is the rate-limiting step in glucose metabolism. It has been reported that some inflammatory cytokines and chemokines, such as TNF-α and interleukin-1, attenuate the activity of insulin-mediated signaling in muscle cells [28,29]. In addition, oxidative stress can also decrease glucose uptake by reducing the activity of insulin-mediated signaling [9,30,31]. In the damaged muscle on the day following the exercise, these cytokines and oxidative components are elevated [32-34], which could lead to the impairment of insulin-dependent glucose uptake. However, because we did not find any significant changes of inflammatory markers and oxidative products in serum and urine, the response is considered to be limited to muscle tissue, rather than the whole body, as suggested in a previous study [5].

Previously, we demonstrated that *Lactobacillus helveticus*–fermented milk attenuates delayed-onset muscle damage after acute exercise in rat [20]. In this study, phagocyte infiltration and inflammatory cytokines expression, markers of inflammation in damaged muscle, were markedly reduced by the consumption of fermented milk. In addition, lipid peroxide levels were elevated after exercise, although the fermented milk uptake significantly reduced this elevation. Therefore, these observations suggest that fermented milk consumption mitigates inflammation and oxidative stress as well as muscle damage, which results in improvements in glucose metabolism by maintaining the insulin signaling pathway. In the present study, we found that ORAC, a marker of antioxidant capacity, was reduced in the placebo trial, but not in the fermented milk trial. Thus, the inhibitory effect of glucose metabolic impairment and muscle damage may be associated with elevated antioxidant levels caused by the consumption of fermented milk. Previously, we demonstrated that, in the skeletal muscle of rats, fermented milk upregulates expression of antioxidant enzymes, such as superoxide dismutase-2, catalase, and glutathione S-transferase α-1 [20]. In addition, heat shock protein 70, a chaperone protein that can function as an antioxidant and an anti-inflammatory agent, was also elevated by the consumption of fermented milk [20]. These observations suggest that fermented milk improves glucose metabolism and muscle damage at least, in part, by controlling endogenous antioxidant and anti-inflammation factors, a hypothesis that is supported by the ORAC results in the present study.

Although the detailed mechanisms of the effects of fermented milk on mitigating muscle damage remain unclear, small peptides present in fermented milk may be the causable agent, because fermented milk is more effective than unfermented milk. Fermented milk is manufactured by fermenting skim milk with a starter culture containing *Lactobacillus helveticus*. During this process, the proteins in skim milk are digested by *Lactobacillus* and converted into small peptides, which are more easily absorbed by the intestines compared to amino acids or large oligopeptides. Such peptides may also have additional physiological benefits aside from their use as a source of protein. Several studies have reported that peptides from fermented milk have various salutary effects, including an antihypertension effect, improvement of arterial stiffness, and immune regulation [12,15,35]. The present study suggests that small digested peptides in fermented milk may contribute to increasing the level of antioxidants in muscle. In future studies, we will attempt to detect the specific small peptides present after the consumption of fermented milk.

## Conclusion

We found that fermented milk prevents glucose metabolic impairment and muscle soreness induced by acute resistance exercise in humans. The reduction of antioxidant capacity was suppressed by the consumption of fermented milk. These observations suggest that dietary fermented milk reduces impairment of glucose metabolism associated with exercise-induced muscle damage via an antioxidant effect. Dietary intake of fermented milk may be useful for persons who perform physical activity for health promotion. In future studies, further research is required to examine the detailed mechanisms of the effect of fermented milk in mitigating muscle damage along with the benefit to athletes.

## Abbreviations

ANOVA: Analysis of variance; CPK: Creatine phosphokinase; ELISA: Enzyme-linked immunosorbent assay; GLUT4: Glucose transporter protein 4; hsCRP: High sensitivity C-reactive protein; ORAC: Oxygen radical absorbance capacity; RM: Repetition maximum; RQ: Respiratory quotient; TNF-α: Tumor necrosis factor alpha; VAS: Visual analog scale; VCO2: Carbon dioxide production; VO2: Oxygen consumption; 8-OHdG: 8-Hydroxydeoxyguanosine.

## Competing interests

The authors declare that they have no competing interests.

## Authors’ contributions

The authors’ contributions were as follows: MI and WA designed the study, analyzed the data, and wrote the manuscript; MI, KM, HY, KF, SS, KT, KH, and SW performed the research; YN and KS discussed results, and WA and AH supervised the overall project. All authors read and approved the final manuscript.

## References

[B1] Aoi W Naito Y Sakuma K Kuchide M Tokuda H Maoka T Toyokuni S Oka S Yasuhara M Yoshikawa T Astaxanthin limits exercise-induced skeletal and cardiac muscle damage in mice Antioxid Redox Signal 2003 5 139 144 10.1089/152308603321223630 12626126

[B2] Aoi W Naito Y Takanami Y Kawai Y Sakuma K Ichikawa H Yoshida N Yoshikawa T Oxidative stress and delayed-onset muscle damage after exercise Free Radic Biol Med 2004 37 480 487 10.1016/j.freeradbiomed.2004.05.008 15256219

[B3] Gissel H Clausen T Excitation-induced Ca^2+^ influx and skeletal muscle cell damage Acta Physiol Scand 2001 171 327 334 10.1046/j.1365-201x.2001.00835.x 11412145

[B4] Proske U Morgan DL Muscle damage from eccentric exercise: mechanism, mechanical signs, adaptation and clinical applications J Physiol 2001 537 333 345 10.1111/j.1469-7793.2001.00333.x 11731568 PMC2278966

[B5] Aoi W Naito Y Tokuda H Tanimura Y Oya-Ito T Yoshikawa T Exercise-induced muscle damage impairs insulin signaling pathway associated with IRS-1 oxidative modification Physiol Res 2012 61 81 88 22188104 10.33549/physiolres.932239

[B6] Del Aguila LF Krishnan RK Ulbrecht JS Farrell PA Correll PH Lang CH Zierath JR Kirwan JP Muscle damage impairs insulin stimulation of IRS-1, PI 3-kinase, and Akt-kinase in human skeletal muscle Am J Physiol Endocrinol Metab 2000 279 206 212 10.1152/ajpendo.2000.279.1.E206 10893341

[B7] De Alvaro C Teruel T Hernandez R Lorenzo M Tumor necrosis factor alpha produces insulin resistance in skeletal muscle by activation of inhibitor kappaB kinase in a p38 MAPK-dependent manner J Biol Chem 2004 279 17070 17078 10.1074/jbc.M312021200 14764603

[B8] Plomgaard P Bouzakri K Krogh-Madsen R Mittendorfer B Zierath JR Pedersen BK Tumor necrosis factor-alpha induces skeletal muscle insulin resistance in healthy human subjects via inhibition of Akt substrate 160 phosphorylation Diabetes 2005 54 2939 2945 10.2337/diabetes.54.10.2939 16186396

[B9] Rudich A Kozlovsky N Potashnik R Bashan N Oxidant stress reduces insulin responsiveness in 3T3-L1 adipocytes Am J Physiol 1997 272 935 940 10.1152/ajpendo.1997.272.5.E935 9176196

[B10] Tidball JG Inflammatory cell response to acute muscle injury Med Sci Sports Exerc 1995 27 1022 1032 10.1249/00005768-199507000-00011 7564969

[B11] Gordon PM Liu D Sartor MA IglayReger HB Pistilli EE Gutmann L Nader GA Hoffman EP Resistance exercise training influences skeletal muscle immune activation: a microarray analysis J Appl Physiol 2012 112 443 453 10.1152/japplphysiol.00860.2011 22052873 PMC3289427

[B12] Nakamura Y Studies on anti-hypertensive peptides in milk fermented with lactobacillus helveticus Bioscience and microflora 2004 23 131 138

[B13] Rebby GV Shahani KM Banerjee MR Inhibitory effect of yoghurt on ehrich ascites tumor cell proliferation J Natl Cancer Inst 1973 50 815 817 4708161 10.1093/jnci/50.3.815

[B14] Takano T Arai K Murota I Hayakawa K Mizutani T Mitsuoka T Effects of feeding sour milk on longevity and tumorigenesis in mice and rats Bifidobact Microflora 1985 4 31 37

[B15] Vinderola G Matar C Perdigón G Milk fermentation products of L. helveticus R389 activate calcineurin as a signal to promote gut mucosal immunity BMC Immunol 2007 8 19 10.1186/1471-2172-8-19 17825099 PMC2045662

[B16] Chapat L Chemin K Dubois B Bourdet-Sicard R Kaiserlian D Lactobacillus casei reduces CD8^+^ T cell-mediated skin inflammation Eur J Immunol 2004 34 2520 2528 10.1002/eji.200425139 15307184

[B17] Nagao F Nakayama M Muto T Okumura K Effects of a fermented milk drink containing lactobacillus casei strain shirota on the immune system Biosci Biotechnol Biochem 2000 64 2706 2708 10.1271/bbb.64.2706 11210142

[B18] Qian B Xing M Cui L Deng Y Xu Y Huang M Zhang S Antioxidant, antihypertensive, and immunomodulatory activities of peptide fractions from fermented skim milk with Lactobacillus delbrueckii ssp. bulgaricus LB340 J Dairy Research 2011 78 72 79 10.1017/S0022029910000889 21214965

[B19] Wang YC Yu RC Chou CC Antioxidative activities of soymilk fermented with lactic acid bacteria and bifidobacteria Food Microbiol 2006 23 128 135 10.1016/j.fm.2005.01.020 16942996

[B20] Aoi W Naito Y Nakamura T Akagiri S Masuyama A Takano T Mizushima K Yoshikawa T Inhibitory effect of fermented milk on delayed-onset muscle damage after exercise J Nutri Biochem 2006 18 140 145 10.1016/j.jnutbio.2006.05.002 16781862

[B21] Drummond MJ Fujita S Abe T Dreyer HC Volpi E Rasmussen BB Human muscle gene expression following resistnce exercise and blood flow restriction Med Sci Sports Exerc 2008 40 691 698 10.1249/MSS.0b013e318160ff84 18317375 PMC5088719

[B22] Frayn KN Calculation of substrate oxidation rates in vivo from gaseous exchange J Appl Physiol 1983 55 628 634 6618956 10.1152/jappl.1983.55.2.628

[B23] Watanabe J Oki T Takebayashi J Yamasaki K Takano-Ishikawa Y Hino A Yasui A Method validation by interlaboratory studies of improved hydrophilic oxygen radical absorbance capacity methods for the determination of antioxidant capacities of antioxidant solutions and food extracts Anal Sci 2012 28 159 165 10.2116/analsci.28.159 22322809

[B24] Cockburn E Robson-Ansley P Hayes PR Stevenson E Effect of volume of milk consumed on the attenuation of exercise-induced muscle damage Eur J Appl Physiol 2012 112 3187 3194 10.1007/s00421-011-2288-2 22227851

[B25] Cartee GD Young DA Sleeper MD Zierath J Wallberg-Henriksson H Holloszy JO Prolonged increase in insulin-stimulated glucose transport in muscle after exercise Am J Physiol 1989 256 494 499 10.1152/ajpendo.1989.256.4.E494 2650561

[B26] Hayashi T Wojtaszewski JF Goodyear LJ Exercise regulation of glucose transport in skeletal muscle Am J Physiol 1997 273 1039 1051 10.1152/ajpendo.1997.273.6.E1039 9435517

[B27] Perseghin G Price TB Petersen KF Roden M Cline GW Gerow K Rothman DL Shulman GI Increased glucose transport-phosphorylation and muscle glycogen synthesis after exercise training in insulin-resistant subjects N Engl J Med 1996 335 1357 1362 10.1056/NEJM199610313351804 8857019

[B28] Lorenzo M Fernández-Veledo S Vila-Bedmar R Garcia-Guerra L De Alvaro C Nieto-Vazquez I Insulin resistance induced by tumor necrosis factor-alpha in myocytes and brown adipocytes J Anim Sci 2008 86 94 104 17940160 10.2527/jas.2007-0462

[B29] Somm E Cettour-Rose P Asensio C Charollais A Klein M Theander-Carrillo C Juge-Aubry CE Dayer JM Nicklin MJ Meda P Rohner-Jeanrenaud F Meier CA Interleukin-1 receptor antagonist is upregulated during diet-induced obesity and regulates insulin sensitivity in rodents Diabetologia 2006 49 387 393 10.1007/s00125-005-0046-x 16385385

[B30] Hansen LL Ikeda Y Olsen GS Busch AK Mosthaf L Insulin signaling is inhibited by micromolar concentrations of H_2_O_2_: Evidence for a role of H_2_O_2_ in tumor necrosis factor alpha-mediated insulin resistance J Biol Chem 1999 274 25078 25084 10.1074/jbc.274.35.25078 10455187

[B31] Rudich A Tirosh A Potashnik R Hemi R Kanety H Bashan N Prolonged oxidative stress impairs insulin-induced GLUT4 translocation in 3T3-L1 adipocytes Diabetes 1998 47 1562 1569 10.2337/diabetes.47.10.1562 9753293

[B32] Ihara H Shino Y Morita Y Kawaguchi E Hashizume N Yoshida M Is skeletal muscle damaged by the oxidative stress following anaerobic exercise? J Clin Lab Anal 2001 15 239 243 10.1002/jcla.1034 11574951 PMC6807904

[B33] Marin DP Dos Santos Rde C Bolin AP Guerra BA Hatanaka E Otton R Cytokines and oxidative stress status following a handball game in elite male players Oxid Med Cell Longev 2011 2011 804873 21922038 10.1155/2011/804873 PMC3172986

[B34] Pedersen BK Ostrowski K Rohde T Bruunsgaard H The cytokine response to strenuous exercise Can J Physiol Pharmacol 1998 76 505 511 10.1139/y98-055 9839076

[B35] Jauhiainen T Rönnback M Vapaatalo H Wuolle K Kautiainen H Groop PH Korpela R Long-term intervention with Lactobacillus helveticus fermented milk reduces augmentation index in hypertensive subjects Eur J Clin Nutr 2010 64 424 431 10.1038/ejcn.2010.3 20145666 PMC2857163

